# To zinc or not to zinc for COVID-19 prophylaxis or treatment?

**DOI:** 10.1099/jmm.0.001299

**Published:** 2021-09-01

**Authors:** Kate Chander Chiang, Ajay Gupta

**Affiliations:** ^1^​ KARE Biosciences, Orange, California, USA; ^2^​ Division of Nephrology, Hypertension and Kidney Transplantation, Department of Medicine, University of California Irvine (UCI) School of Medicine, Irvine, California, USA

**Keywords:** COVID-19, copper, interferon, nutritional deficiency, prophylaxis, zinc

Dear Editor,

We read with interest the recently published retrospective, observational study by Carlucci and colleagues examining zinc as a determinant of outcomes in hospitalized coronavirus disease 2019 (COVID-19) patients [1]. The authors found an association between improvement in certain outcomes when COVD-19 patients were treated with a combination of zinc, hydroxychloroquine and azithromycin, compared with treatment with hydroxychloroquine and azithromycin. However, as pointed out in a recent review of the study by Gbinigie and Akyea *et al*., the observed associations provide information about zinc in combination with other medications but not as a standalone treatment [2]. Notably, Carlucci and colleagues failed to find a benefit of zinc supplementation in severe COVID-19 patients requiring intensive care unit (ICU) care. Even though conclusive evidence is lacking, it is commonly believed that zinc may be beneficial in reducing the symptoms and duration of upper respiratory viral illnesses, including the common cold [3]. However, any beneficial effects of zinc supplementation in cases of severe acute respiratory syndrome coronavirus 2 (SARS-CoV-2) infection may be attributable to correction of subclinical zinc deficiency rather than the supraphysiological levels achieved in zinc-replete subjects [4]. Unfortunately, this cannot be determined from the Carlucci study since baseline zinc levels were not reported. Even though zinc has been shown to inhibit viral RNA-dependent RNA polymerase of SARS-CoV *in vitro* [5], there are no clinical studies of zinc for SARS-CoV 2003. While expected to be similar to SARS-CoV, the effect of zinc on SARS-CoV-2 RNA polymerase has not been reported to the best of our knowledge. In the interim, pending the results of ongoing randomized controlled clinical trials such as HELPCOVID-19 and ZnD3-CoVici, we suggest abundant caution when prescribing zinc for prophylaxis or treatment of COVID-19 for the reasons outlined below.

SARS-CoV-2 is generally transmitted by aerosol and possibly by the faecal–oral route [6]. Epithelial cells that comprise mucosal barriers in the respiratory tract or the gut produce interferons (IFNs), especially type III IFNs, as important components of the host innate antiviral response at mucosal barriers. Type III IFN, comprising IFN-λ1, 2, 3 and 4, bind to interferon IFNL receptor 1 on epithelial cells in order to mount antiviral defence [7, 8]. Zinc can bind to interferon IFNL receptor 1, thereby blocking type III interferon (IFN) antiviral responses and interfering with the barrier function of respiratory and intestinal mucosa. In fact, in hepatitis C infection, higher zinc levels were associated with near doubling of viral load, putatively by inhibiting type III IFN action [8]. Amongst interferons, IFN-λ2 and IFN-λ3 serve as a potent defence against SARS-CoV-2 [7]. A deficient IFN-λ host response could facilitate viral replication in the upper respiratory tract, with spread of the virus down the airways and into the lungs, while promoting spread of the virus to contacts [7]. Thus, in early infection, at the upper respiratory mucosal barrier, it is unclear if supplemental zinc would help or hurt viral attack. Later in the course of infection, Broggi *et al*. reported that morbidity in COVID-19 patients correlates with high expression of type I and III IFNs in the lung, and that IFN-λ secreted by dendritic cells in the lungs causes damage to the lung epithelium, which increases susceptibility to bacterial superinfections [7]. Similarly, Major *et al.* found that IFN-λ hampers lung repair by inducing p53 and inhibiting epithelial proliferation and differentiation in a mouse model of influenza infection [9]. The results of these studies suggest that the location, timing and duration of IFN exposure and therefore zinc treatment are critical parameters underlying the success or failure of zinc in viral respiratory infections, including SARS-CoV-2.

Zinc, although essential for activity of many enzymes, transcription factors and signaling molecules, can be cytotoxic per se, inducing apoptosis in T cells, B cells and thymocytes [10, 11]. Incubation of peripheral blood mononuclear cells with zinc *in vitro* led to release of cytokines such as IL-1, IL-6, TNF-α and IFN-γ [12]. Zinc suppresses natural killer cell killing and T-cell functions while activating monocytes and in higher concentrations stimulating chemotaxis of neutrophil granulocytes [12]. Severe COVID-19 disease is characterized by a maladaptive immune response comprising dysregulation of myeloid cell compartments [13], neutrophil extracellular trap release [14] and lymphopenia [15]. Therefore, zinc supplementation in zinc-replete patients has the potential to further aggravate the maladaptive innate and adaptive immune responses to SARS-CoV-2 infection. Zinc also serves as an intracellular signaling molecule [10]. Activation of Toll-like receptors induces a decrease in intracellular free zinc, which activates dendritic cell response [10, 16]. It is noteworthy that TLR4-mediated inflammatory signaling is upregulated in COVID-19 patients and this is regulated by zinc [17]. Increase in extracellular zinc can induce an increase in intracellular zinc, referred to as a zinc wave [10], and this can interfere with TLR4-mediated dendritic cell maturation [10], a critical component of immune response to SARS-CoV-2 infection [13].

It has recently been recognized that a maladaptive type 2 immune response normally triggered by helminths is associated with severity of COVID-19 disease [18]. Mast cells play a key role in type 2, T helper 2-mediated immune response in COVID-19 [19]. Mast cell activation is triggered by a perinuclear intracellular zinc wave mediated by various stimuli, including immunoglobulin E, that trigger inhibition of phosphatase activity and modulation of mitogen-activated protein kinase activation, thereby increasing expression of interleukin-6 (IL-6) and TNF-α [10]. IL-6 and TNF-α are biomarkers of severe COVID-19 [20], and upregulation of these biomarkers by zinc may explain the lack of benefit of zinc in severe COVID-19 cases observed by Carlucci *et al*.

Furthermore, zinc supplements, if consumed long-term for chemoprophylaxis, can interfere with absorption of dietary copper by competing with copper at the intestinal brush border during the luminal phase of absorption, thereby inducing copper deficiency [21]. Copper deficiency leads to anaemia, leukopenia and immune suppression [21]. Copper is also a potent antioxidant [21], and copper deficiency could exacerbate oxidative stress in COVID-19. The population reference intake (PRI) for zinc is 7.2 to 12.7 mg/day for women and 9.4 to 16.3 mg/day for men [22]. However, relatively inexpensive supplements containing up to 50 mg elemental zinc per unit dose are readily available online or over the counter, thereby facilitating possible over supplementation in COVID-19 cases. Paracelsus, the 15th century physician who pioneered the use of chemicals and minerals in medicine, expressed the classic toxicology maxim ‘All things are poison, and nothing is without poison; the dosage alone makes it so a thing is not a poison’ [23]. Therefore, the presence or absence of zinc deficiency in the host, the dose of zinc and the duration of treatment are critical considerations when prescribing zinc in COVID-19 cases.

In conclusion, zinc administration in COVID-19 patients can influence the immune system positively by correcting any underlying zinc deficiency; and potentially negatively in zinc-replete patients by interfering with interferon lambda response, causing cytotoxicity to T and B cells and impairing TLR-4-mediated dendritic cell maturation. Randomized controlled trials are urgently needed to test the efficacy and safety of zinc in patients with COVID-19, and in the interim prudence is warranted when prescribing or consuming zinc supplements for prophylaxis or treatment of COVID-19.
